# Oral manifestations associated with inflammatory bowel disease and early endoscopic findings in patients with spondyloarthritis

**DOI:** 10.1186/s12903-022-02497-4

**Published:** 2022-11-09

**Authors:** Andrés Alvarado-Julio, Katherin Chumacero-Palma, María Rosa Buenahora, Viviana Parra-Izquierdo, Mónica Monsalve, Ana María Torres, Lorena Chila-Moreno, Cristian Flórez-Sarmiento, Alejandro Ramos-Casallas, Juliette De Avila, Juan Manuel Bello-Gualtero, Diego Jaimes, Adriana Beltrán-Ostos, Philippe Chalem-Choueka, César Pacheco-Tena, Wilson Bautista-Molano, Consuelo Romero-Sánchez

**Affiliations:** 1 Oral Pathology and Diagnostic Media, School of Dentistry, Universidad El Bosque, Av. Carrera 9 #131A-02, Bogotá, Colombia; 2 Clinical Epidemiology Unit/UNIECLO, School of Dentistry, Universidad El Bosque, Av. Carrera 9 #131A-02, Bogotá, Colombia; 3 School of Dentistry, Cellular and Molecular Immunology Group/INMUBO, Universidad El Bosque, Av. Carrera 9 #131A-02, Bogotá, Colombia; 4 Gastroadvanced SAS IPS, Carrera 23 #45C-31, Bogotá, Colombia; 5 Rheumatology and Immunology Department/Clinical Immunology Group, Hospital Militar Central, Transversal 3ª #49-00, Bogotá, Colombia; 6 School of Medicine, Clinical Immunology Group, Universidad Militar Nueva Granada/Hospital Militar Central, Transversal 3ª #49-00, Bogotá, Colombia; 7 Clínicos IPS, Carrera 15 #98-29, Bogotá, Colombia; 8 Fundación Instituto de Reumatología Fernando Chalem, Calle 73 #20A - 27, Bogotá, Colombia; 9 Investigación Y Biomedicina De Chihuahua S.C., Calle 16 #1600, Chihuahua, CHIH México

**Keywords:** Ankylosing spondylitis, Aphthous stomatitis, Gingivitis, Inflammatory bowel disease, Spondyloarthritis

## Abstract

**Background and aims:**

Spondyloarthritis (SpA) is a group of autoinflammatory disorders, of which the primary extra-articular manifestation is inflammatory bowel disease (IBD). The oral cavity being a part of gastrointestinal tract, is significantly compromised in IBD, and in many cases, it is the first site of clinical manifestations of IBD. This study aimed to identify changes in the oral mucosa associated with the onset of IBD and their association with endoscopic/histological findings.

**Materials and methods:**

The study assessed 80 patients with SpA and 52 healthy controls. Oral, rheumatological, and gastroenterological assessments were performed. The ileocolonoscopy was performed via digital magnification chromoendoscopy. The statistical analysis consisted of Chi-square, Fisher's exact, and multiple correspondence discriminant analysis tests.

**Results:**

From the disease cohort, 63.0% patients showed oral lesions (*p* = 0.050). These manifestations ranged from gingivitis (55.0%, *p* = 0.001), aphthous stomatitis (3.8%, *p* = 0.091), angular cheilitis (2.6%, *p* = 0.200), and perioral erythema with scaling (1.3%, *p* = 0.300). All patients who presented with alterations in colonic mucosa also had oral lesions associated with IBD (*p* = 0.039), specifically gingivitis/aphthous stomatitis (*p* = 0.029).

**Conclusion:**

The patients with SpA without IBD present significant oral signs and symptoms. Gingivitis seems to be the most relevant because of its associations with early endoscopic and histological findings.

**Clinical relevance:**

An integral approach to the diagnostic tests that includes evaluations of oral, rheumatological and gastroenterological tissues may favor timely attention and improve patients’ quality of life.

**Supplementary Information:**

The online version contains supplementary material available at 10.1186/s12903-022-02497-4.

## Introduction

The concept of spondylarthritis (SpA) proposed by the Assessment of Spondyloarthritis International Society (ASAS) currently refers to a heterogeneous group of chronic autoinflammatory rheumatic disorders or changes [1]. Several subtypes include ankylosing spondylitis (AS) as the prototype. Other forms of SpA are juvenile spondylarthritis, spondylarthritis associated with psoriatic arthritis, inflammatory bowel disease (IBD), and reactive arthritis (ReA). Manifestations that align with the abovementioned forms of disease to some extent but cannot be classified into a specific form, are called “undifferentiated spondyloarthritis” (undifferentiated SpA). According to this criteria, this condition is classified into two large groups: peripheral spondyloarthritis (pSpA) and axial spondyloarthritis (AxSpA) [2].

SpA is associated with extra-articular presence of uveitis, arthritis, enthesitis, dactylitis, psoriasis, and intestinal inflammation which can range from a subclinical alteration to established IBD [3]. IBD is a complex disease characterized by chronic inflammation of the gastrointestinal tract mucosa, resulting from the interaction of several factors, including genetic and environmental, and host adaptive immunity. It has been classified into two phenotypes: ulcerative colitis (UC) and Crohn's disease (CD). CD is characterized by its ability to affect any part of the gastrointestinal tract in a discontinuous manner, and it is often transmural and granulomatous. On the other hand, UC tends to affect the rectum and adjacent colon mucosa continuously, affecting only the mucosa and submucosa [4, 5].

The frequency of gastrointestinal manifestations in SpA ranges from 21.0 to 30.0%, with a wide clinical spectrum. Approximately 5.0–10.0% of these symptoms are associated with IBD. A substantial percentage of patients with nonspecific gastrointestinal symptoms may have subclinical intestinal inflammation that can be confirmed only via endoscopy and histology. However, this confirmation may be delayed in many cases [6, 7].

The incidence of IBD has been increasing progressively and on a global scale. Although IBD primarily affects the intestinal tract, extraintestinal manifestations of the disease are observed in 50% of the patients, particularly in the oral cavity, which can be compromised in both UC and CD. Some specific oral manifestations in patients with CD included: indurated mucosal tags, cobblestone-like lesions and mucogingivitis, deep linear ulcerations, and lip inflammation with vertical fissures [8]. The pathology of CD in upper GI track is more prevalent in children and adolescents, indicative of worse prognosis and perianal changes [9]. In some cases, early onset screening for gastrointestinal disorders with oral manifestations may motivate patients toward a timely consultation and obtain regular endoscopic and histological evaluations, thus improving their quality of life.

To the best of our knowledge, no previous studies evaluated the oral mucosal changes associated with IBD in patients with SpA. In some cases, proactive screening for gastrointestinal disorders with early oral manifestations may guide patients toward a timely consultation at the gastroenterology department as well as endoscopic and histological evaluations, thus improving their quality of life.

Based on the abovementioned information, this study sought to identify changes in the oral mucosa, (clinicopathological manifestations associated with IBD), in patients with SpA without a confirmed IBD diagnosis and their association with endoscopic and histological findings.

## Materials and methods

This descriptive observational study assessed oral clinical manifestations such as oral lesions due to changes in the oral mucosa as well as the results of the clinical rheumatological, endoscopic/histological evaluations in patients with SpA.

### Patients

In each institution was a work team with 2 or 3 rheumatologists. Subsequently, experts selected 409 medical records of patients preliminarily classified by the participating institutions with a diagnosis of SpA. In total, 240 patients fulfilled the meeting criteria according to the European Spondyloarthropathy Study Group (ESSG) and the classification criteria of the Assessment of Spondyloarthritis International Society (ASAS) [1, 10, 11]. Later, experts selected the patients and performed the respective clinical review and inquiring about the presence of two or more gastrointestinal symptoms during the last 6 months [12, 13]. Of these patients, 80 were selected. Gastroenterologists attended the clinical consultation, performed the ileocolonoscopy with chromoendoscopy and digital magnification procedure, and interpreted the pathology results. Oral pathologists evaluated the patients according to the modified World Health Organization guidelines. In each institution and each area, only one expert was the final gold standard for the selection process.

### Inclusion criteria for patients with SpA

This study included individuals aged 18–65 years who met the SpA clinical diagnosis criteria [1, 10, 11]. Patients were interviewed, and their anamnesis was reviewed to determine the presence of gastrointestinal symptoms (i.e., diarrhea, mucus stools, hematochezia, daily stool number, and abdominal pain and distention); musculoskeletal symptoms (i.e., lower back pain, arthritis, dactylitis, and enthesitis); history of infection, uveitis, psoriasis, or IBD; and presence of the HLA-B27 allele. Furthermore, the time of symptom and disease progression was calculated using the Bath Ankylosing Spondylitis Disease Activity Index (BASDAI) and Ankylosing Spondylitis Disease Activity Score (ASDAS), and C-reactive protein (CRP)/erythrocyte sedimentation rate (ESR) [14, 15].

### Exclusion criteria for patients and controls

The following exclusion criteria were used: pregnancy, use of antibiotics during the last 3 months, history of infection during the last month, diagnosis of another autoimmune disease, neoplasia, immunodeficiency, chronic pancreatitis or liver disease, diabetes, and having received any dose of systemic steroids at the time of inclusion in the study and those patients with concomitant SpA and IBD.

### Inclusion criteria for controls

The control group was selected in healthy individuals with an age range between 18 to 65 years. with lifestyles, socioeconomic status, and professions similar to those of the patients were included.

They were reviewed by rheumatologists and gastroenterologists, and they were questioned regarding the presence of gastrointestinal symptoms (i.e., diarrhea, stools with mucus, hematochezia, daily stool number, and abdominal pain and distension); oral clinical examination was also performed. Finally, they were evaluated for serological and fecal inflammation parameters.

Only some control individuals were evaluated endoscopically since they were undergoing a general clinical check-up and had a clinical indication for this procedure. Ethically, an invasive procedure such as colonoscopy should not be performed in individuals who do not require it.

### Tabulation and analysis strategy

The following assumptions were made to calculate the sample size: an estimated proportion of colonic mucosa changes in SpA associated with the disease activity index (odds ratio [OR] = 1.94, *p* = 0.009) and a confidence interval with a width equal to twice the accepted error (20.0%) with a confidence level of 95% for a sample size of at least 77 patients with SpA, with a power of 0.8 and type II error of 0.2.

The healthy controls and patients were matched based on age (± 2 years). Comparative analyses were performed using chi-square, Fisher's exact, and multiple correspondence discriminant analysis tests [16]. Multiple correspondence discriminant analysis allows for grouping variables with high correlation coefficients (CCs) and discriminating groups of patients with common characteristics. The results are shown on a Cartesian plane, where the variables are represented as vectors whose angles become more acute as the level of correlation between them increases. The length of each vector represents the CC of the variable within the group, which ranges from − 1.0 to + 1.0. A high contribution was considered when CC values were > 0.7, intermediate when 0.5–0.7, and low when < 0.5. All variables with CCs < 0.3 were excluded.

### Evaluation of the oral mucosa

All participants were evaluated by oral pathologists according to the modified World Health Organization guidelines [17]. Chain ganglia and cervicofacial muscles were evaluated in the extraoral examination.

Various anatomic sites of the oral cavity evaluated during the clinical examination included: lips, jugal mucosa, gums, hard and soft palates, oropharynx, all surfaces of the tongue, floor of the mouth and individual tooth count. Gingivitis diagnostic criteria were transudate of gingival fluid, redness of the gingival margin, loss of rough gingival texture and bleeding by touching the gingival margin with a blunt instrument [18].

Datasets were collected through a form that provided general patient data, extraoral (cervical, facial and articular) and intraoral exam findings.

An analysis was performed to determine the number of lesions in the abovementioned areas that correspond to specific elemental lesion (macula, papule, plaque, gallbladder, bulla, erosion, ulcer or nodule), associated lesion (angular cheilitis, perioral erythema with scaling, aphthous stomatitis, gingivitis, periodontal disease or dental caries), or other forms that are not mentioned, emphasizing their location and number. The clinical indexes were taken by pathologists in each institution who participated in an inter-examination calibration vs gold standard. In addition, repeated evaluations were conducted before the study on 5 randomly selected subjects in order to determine intra-examiner reproducibility. Only those with correlation indexes greater than 0.90 participated.

In all phases of the research, the tenets of respect for the dignity and protection of participants’ rights and well-being were upheld. The protocol, data collection format, and study procedure were previously reviewed and approved by the Institutional Research Ethics Committee of Hospital Militar Central, Bogotá, Colombia (approval number: HMC 2017-023).

### Digital chromoendoscopy colonoscopy with narrow-band imaging

A clinical evaluation of the patients was performed at the gastroenterology department [19]. The importance, benefits and risks of a colonoscopy procedure were explained to them. After obtaining the patient’s informed consent, low-volume Travad Pik ® (Cali, Colombia) preparation was performed before colonoscopy (sodium picosulfate: 10 mg + light magnesium oxide: 3.5 g + citric acid: 12 g). This allowed for an adequate cleaning of all the colonic and distal ileum tracts, measured with Boston 9/9 scale [20].

During colonoscopies, the patients were sedated with intravenous propofol by an anesthesiologist and the procedures were performed by a gastroenterologist expert in diagnostic and therapeutic endoscopy [21]. The Olympus EVIS EXERA III (CF-HQ190L/I; Tokyo, Japan) or FUJI EC-760ZP-V / L Zoom, ELUXEO 700 Series (Tokyo, Japan) equipment was used. These devices can perform electronic magnification of the mucosa, digital narrow-band imaging (Olympus) and blue-light imaging (FUJI, Tokyo, Japan) chromoendoscopic evaluations. The imaging captured submucosal vascular pattern in the ileum and colon, and the pattern of the colonic crypts. Additionally, the procedure included taking biopsies of distal ileum, left colon, rectosigmoid, and other sites where any abnormality was observed by magnification and digital chromoendoscopy.

### Histological evaluation

The traditional technique of paraffin-embedding, fine cutting, and routine staining with hematoxylin–eosin was used before the microscopic evaluation of the samples obtained (distal ileum, sigmoid colon, and rectum). On average, 18–24 per block were obtained from each sample, and when necessary, more levels were made. Special staining (Masson's trichrome, Gomory, PAS with and without diastase, Ziehl–Neelsen) or immunohistochemical analyses were performed as needed in corresponding cases and according to the diagnosis. The procedure was evaluated by an experienced professional pathologist [22].

## Results

### Sociodemographic and clinical characteristics of patients with SpA

This study included 80 patients with SpA: 56.0% men with a mean age of 42.8 (standard deviation [SD]: ± 10.4; range 35–50) years and a mean body mass index (BMI) of 25 (range 23.9–28.4 kg/m^2^). Of the 52 healthy controls, 54.0% were men with a mean age of 41 (SD: ± 13.6; range 30–53) years and a mean BMI of 25.1 (range 22.9–27.6 kg/m^2^; Additional file 1: Table S1).

**Fig. 1:**
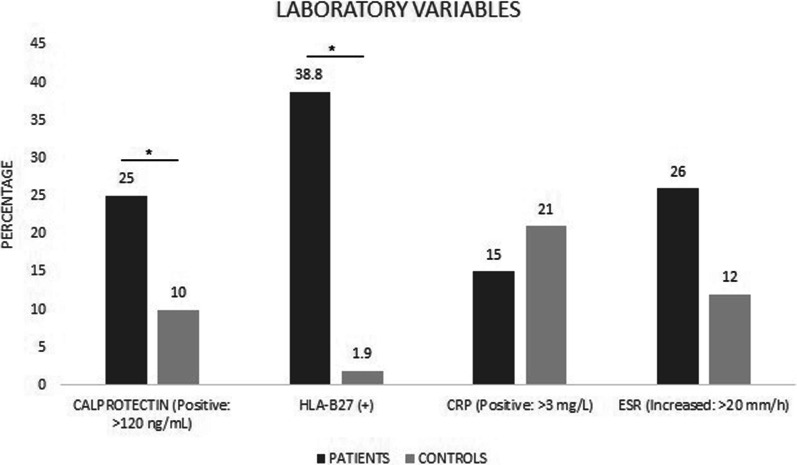
Frequency of positivity for each inflammation-related variable in the total group of patients with spondyloarthritis and healthy controls. HLA-B27: human leukocyte antigen-B27, CRP: C-reactive protein, and ESR, erythrocyte sedimentation rate. The following tests were used for the analysis of statistical significance: chi-square, Fisher's exact, Wilcoxon's rank-sum tests. *Statistical significance (*p* < 0.05)

Among the patients with SpA, 6.2% smoked, 31.0% reported a smoking history, and 15.0% were passive smokers. Most of them were employed (41.0%), owned a house (51.0%), were married (56.0%), and were professionals (49.0%). Of the healthy controls, 15.0% smoked, 31.0% had a smoking history, and 21.0% were passive smokers. Most of them were employed (77.0%), owned a house (67.0%), were single (40.0%), and were professionals (54.0%; Additional file 1: Table S1). The laboratory findings in the total group of SpA patients revealed that 38.8% of patients were positive for the HLA-B27 allele with statistical significance (*p* = 0.001; Fig. 1). In most patients, the disease status was reported as high disease activity (BASDAI ≥ 4: 52.0%; ASDAS-CRP ≥ 2.1: 74.3%; and ASDAS-ESR ≥ 2.1: 94.0%).

### Rheumatological clinical variables in patients with SpA

Of the patients with SpA, 82% were diagnosed with AS; on average, the time of disease symptomatology was 9.2 (SD: ± 5.9) years. The patients were classified according to ASAS criteria as compromised AxSpA (81.0%), pSpA (20.0%) or with both axial and peripheral symptoms (56.0%). All patients reported at least one musculoskeletal symptom, the most frequent ones being inflammatory lumbar pain (80.0%), enthesitis (71.0%), and arthritis (68.0%). Finally, 11.0% patients presented with a previous infection and 59.0% suffered from fatigue. No associations were noted between the rheumatological variables and oral manifestations.

The pharmacological management of the study group was: 60% received biological treatment and 40% conventional management, of all the patients 26.3% received anti-inflammatory drugs, 2.5% used Methotrexate, Sulfasalazine 3.8%. Faced with the use of IL-17 inhibitors, 7.3% of patients receive this management and respect to anti-TNF, it was found that 44 patients are receiving this therapy for 54.9% of all those studied.

### Gastrointestinal manifestations in the group of patients with SpA

**Fig. 2:**
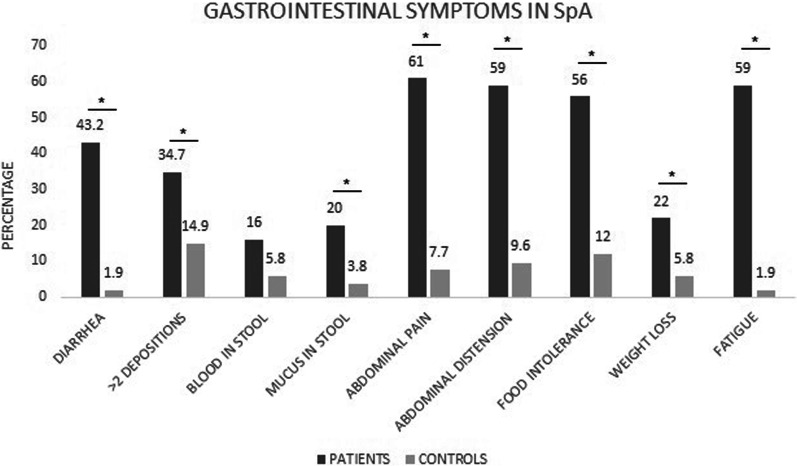
Percentage of positivity of gastrointestinal clinical manifestations in patients with spondyloarthritis and healthy controls. The following tests were used for the analysis of statistical significance: chi-square and Fisher's exact tests. *Statistical significance (*p* < 0.05)

From the patient pool, 69.0% reported as compared to 7.7% from health control group highlighting a statistically significant difference (*p* = 0.001; Fig. 2). In terms of individual signs and symptoms, there was a significant difference observed when evaluating for presence of diarrhea, abdominal pain (*p* = 0.001), mucus in stool (*p* = 0.017) and weight loss (*p* = 0.020). However, the report of blood in stool was not significantly different between the two cohorts (*p* = 0.130; Fig. 2).

Further, comparing the inflammation markers, calprotectin concentration in stools of 25.0% SpA patients was > 120 ng/ml as compared to detectable levels observed only in 10.0% of healthy controls (*p* = 0.056, Fig. 2).

### Endoscopic and histological findings

From the total of 80 patients, 41 underwent an ileocolonoscopy endoscopic study with chromoendoscopy and digital magnification including sample collection for pathologic tests. Of them, 17.1% had the following changes in the rectal mucosa: erythema (4.1%), vascular pattern loss (17.1%), erosion (9.8%), ulcer (2.4%), and rectal inflammation (2.4%).

**Fig. 3:**
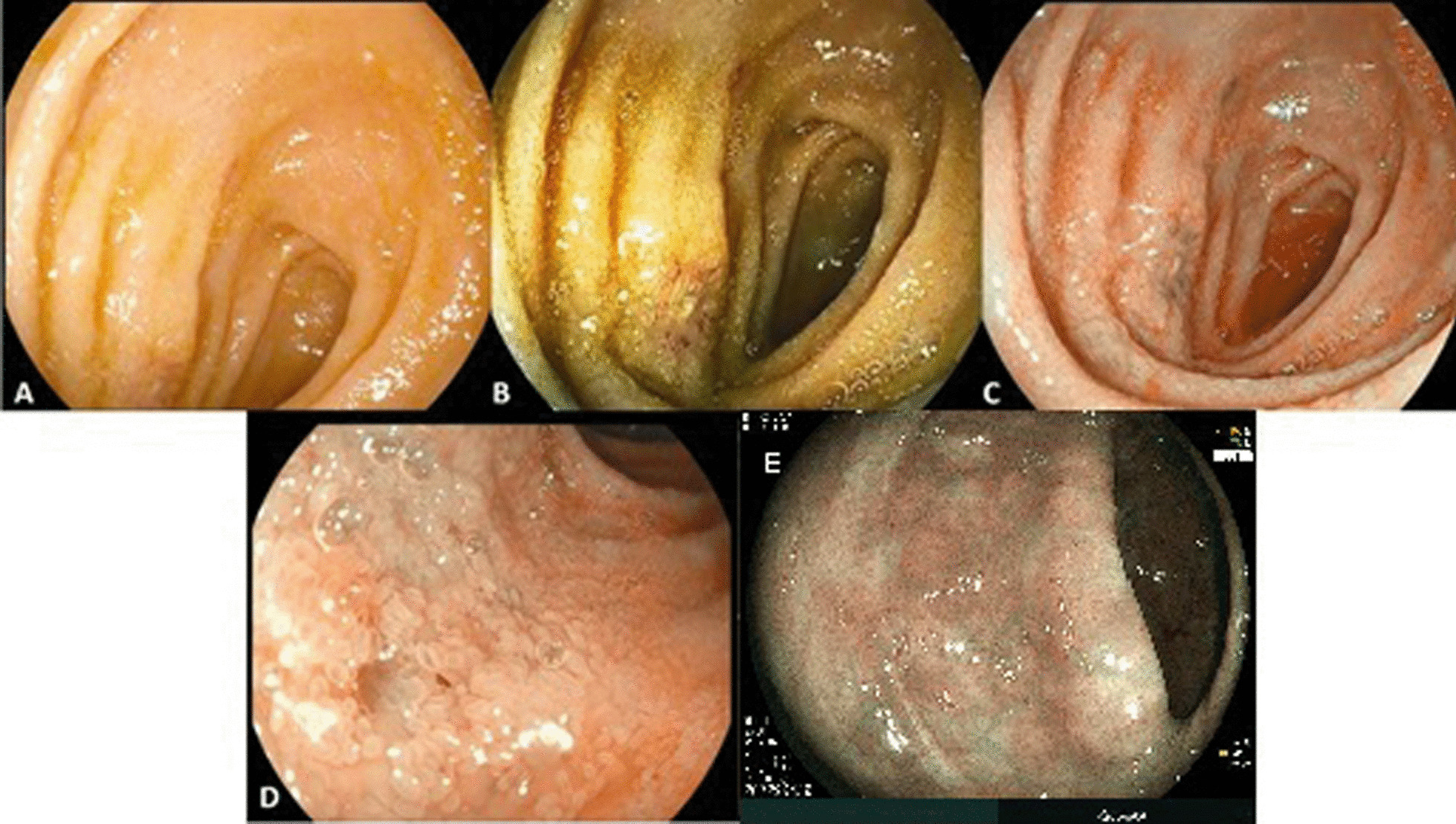
Endoscopic findings in patients with spondyloarthritis. **A** Distal ileum, decrease in the size of the villi. **B** Digital chromoendoscopy (I-San 2), ileum ulcers with the loss of villi. **C** Digital chromoendoscopy (I-San 3), ileal ulcers with the loss of villi. **D** Digital chromoendoscopy (I-San 3) with magnification, denuded areas in the ileum with the loss of villi. **E** Digital chromoendoscopy (I-San 3) with magnification, attenuation of vascular pattern in colon with mild distortion of colonic crypts

In the evaluation of the sigmoid colon, changes in the mucosa were observed in 17.1% patients, erythema in 2.1%, vascular pattern loss in 9.8%, erosion in 9.8%, and ulcer in 2.4%. Mucosal changes in the ileum were observed in 41.5% of patients, erythema in 9.8%, vascular pattern loss in 29.3%, erosion in 9.8%, and ulcer in 12.2%. Atrophy of the villi was noted in 36.6% patients and hemorrhoids in 22.0% (Fig. 3).

At the ileal level, the histopathological analysis and diagnosis showed an inflamed pattern in the mucosa of 41.5% patients and morphological changes in tissues of 31.7% patients with presence of rectal bleeding (*p* = 0.034). Cryptitis or ileal atrophy (24.4%), chronic inflammation (31.7%) and acute inflammation (12.2%) were reported in SpA patients.

Microscopic studies of the colon revealed an inflammatory pattern in 31.7% patients, chronic inflammation in 26.8%, and acute inflammation in 17.1%. Pathologic changes were noted in the colonic submucosa of 2.4% patients, and findings such as cryptitis or atrophy of the colonic villi were detected in 14.6%. Finally, rectal inflammation was reported in 19.5% patients and eosinophilia in 4.9%. Thus, a significant trend was found between the presence of a chronic inflammatory pattern and diarrhea (*p* = 0.060).

### Early inflammatory and structural clinical changes in the oral mucosa of patients with SpA

#### IBD-associated changes

**Fig. 4:**
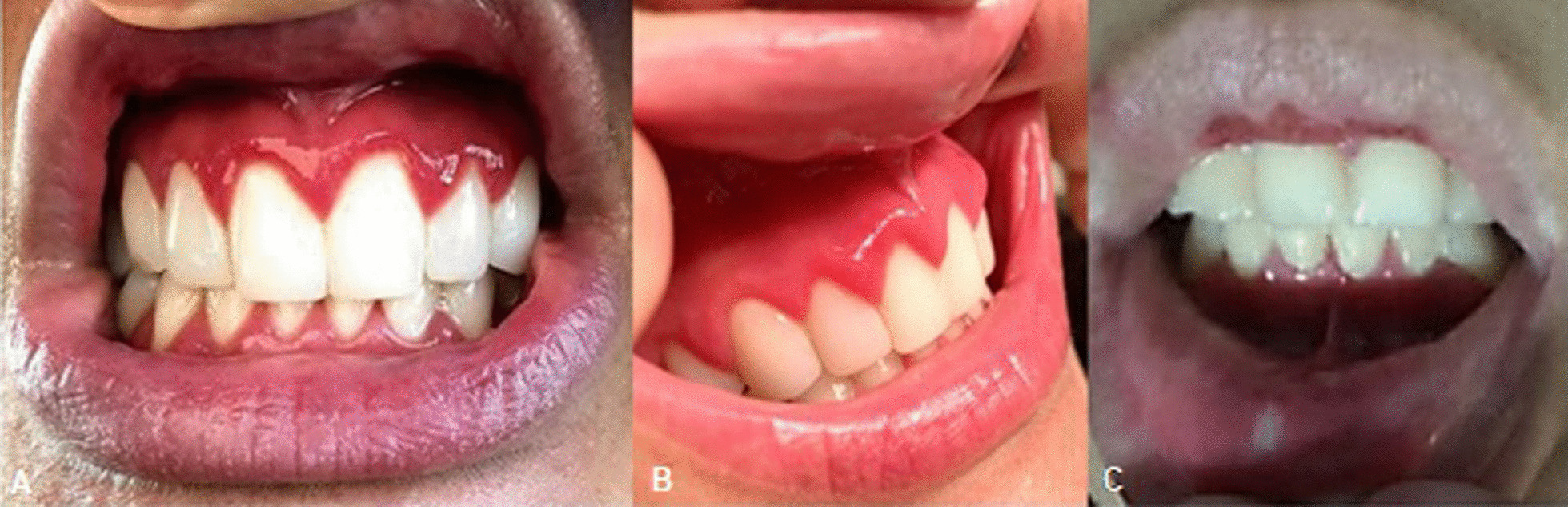
Oral clinical manifestations in patients with spondyloarthritis. **A** Erythematous gums with enlarged interproximal dental papillae of generalized presentation compatible with the diagnosis of gingivitis. Common nonspecific oral manifestations associated with inflammatory bowel disease (IBD). **B** Multiple minor ulcerated lesions compatible with aphthous stomatitis and spontaneous bleeding from the gums, indicating the diagnosis of gingivitis—common nonspecific oral manifestation associated with IBD

Structural and inflammatory changes in the oral mucosa characterized by the presence of oral lesions were observed in 63.0% patients. Most common pathologies included: gingivitis (55.0%), aphthous stomatitis (3.8%), angular cheilitis (2.6%), and perioral erythema with desquamation (1.3%; Fig. 4). Among the healthy controls, 44.0% developed oral lesions such as gingivitis (42.0%) and candidiasis (2.0%, Table 1).

**Table 1:** Oral manifestations in patients with spa and healthy controls

Characteristics	Patients (N = 80)		Healthy controls (N = 52)		*P* value
	n	%	n	%	
Oral manifestation					
Inflammatory lymph node manifestation	5	78.0	1	2.0	0.900
Oral lesions associated with IBD					0.050†
Present	29	37.0	28	56.0	
Nonpresent	49	63.0	22	44.0	
Indeterminate	2				
Type of associated lesion					
Angular Cheilitis	2	2.6	0	0.0	0.200
Perioral erythema with scaling	1	1.3	0	0.0	0.300
Aphthous stomatitis	3	3.8	0	0.0	0.091
Gingivitis	43	55.0	21	42.0	0.001†
Candidiasis	0	0.0	1	2.0	0.300
Indeterminate	2				
Oral lesions nonassociated with IBD					0.700
Nonpresent	58	74.0	39	81.0	
Physiological pigmentation	2	2.6	3	6.2	
Traumatic ulcer	7	9.0	2	4.2	
Traumatic fibroma/IFH	2	2.6	1	2.1	
Leukoedema	1	1.3	0	0.0	
Subprosthetic stomatitis	1	1.3	2	4.2	
Nicotinic stomatitis	1	1.3	0	0.0	
Palatine/lingual torus	3	3.8	0	0.0	
Amalgam tattoo	1	1.3	0	0.0	
Mucocele	1	1.3	0	0.0	
Traumatic petechiae	1	1.3	0	0.0	
Leukoplakia	0	0.0	1	2.1	
Indeterminate	2		4		

IBD, inflammatory bowel disease; PD, presumptive diagnosis; IFH, inflammatory fibrous hyperplasia

^†^Statistically significant difference (Fisher exact test)

#### IBD-nonassociated changes

On the basis of presumptive clinical diagnosis, oral lesions not associated with IBD included traumatic ulcer (9.0%), palatine/lingual torus (3.8%), racial melanosis traumatic fibroma (2.6%), and leukoedema, subprosthetic stomatitis, nicotinic stomatitis, amalgam tattoo, mucocele, and petechiae due to trauma (1.3%). The most common lesions in the healthy controls were racial pigmentation (6.2%), subprosthetic stomatitis and traumatic ulcer (4.2%; Table 1).

### Comparison of gastrointestinal manifestations between patients with SpA and healthy controls

In patients with SpA, 69.9% reported the presence of some signs and symptoms of gastrointestinal origin, as compared to healthy controls (7.7%; *p* = 0.001). Furthermore, the presence of diarrhea for > 4 weeks, abdominal pain, bloating, fatigue, and food tolerance was statically different between the two groups (*p* = 0.001, Fig. 2).

Furthermore, 38.8% patients were positive for the HLA-B27 allele, whereas this percentage was 1.9% for the healthy controls, with statistically significant differences (*p* = 0.001).

### Comparison between clinical and structural changes in the oral mucosa of healthy controls and patients

#### SpA and IBD-associated gastrointestinal manifestations

**Fig. 5:**
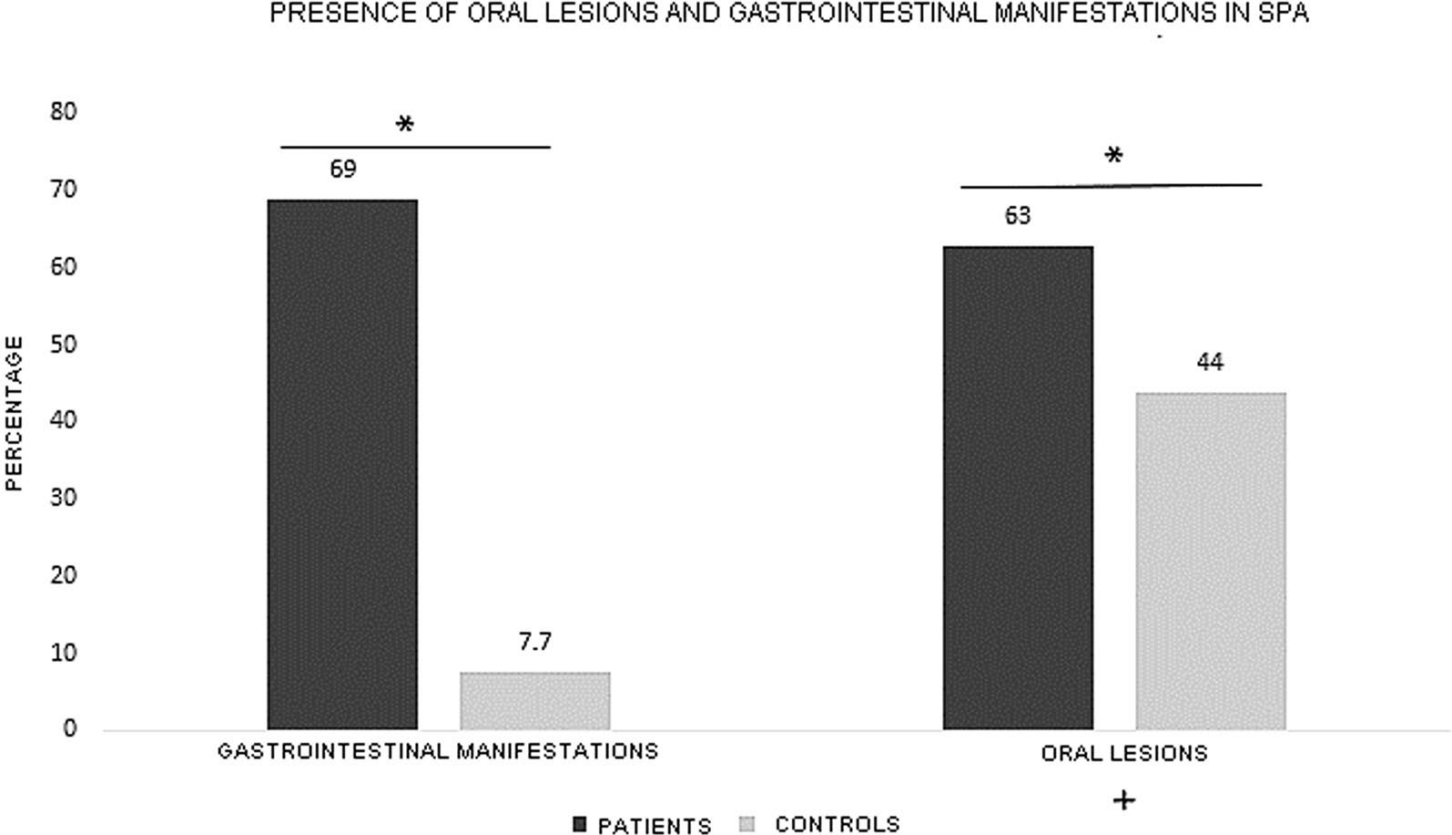
Presence of oral and gastrointestinal changes in patients with spondyloarthritis compared with healthy controls. The following tests were used for the analysis of statistical significance: chi-square, Fisher's exact, and univariate logistic regression tests. Significant difference was observed between the two groups. *Statistical significance (*p* < 0.05)

There was a significant difference in the presence of clinically inflamed oral mucosa in SpA patients as compared to healthy controls (63.0% vs. 44.0% respectively; *p* = 0.050; Fig. 5).

#### SpA and IBD-non-associated gastrointestinal manifestations

Oral lesions not associated with IBD included: traumatic ulcer, palatal/lingual torus, racial pigmentation and traumatic fibroma. The most common lesions in healthy controls were racial pigmentation, subprosthetic stomatitis, and traumatic ulcer. There were no significant differences in the frequency of these lesions.

### Association between oral manifestations and endoscopic and histological findings

When analyzing the findings of the colonoscopies with digital chromoendoscopy and magnification of the sigmoid colon mucosa, 100.0% of the patients with changes in colonic mucosa had some IBD-associated oral lesions (*p* = 0.039), most commonly gingivitis and aphthous stomatitis (*p* = 0.029). When analyzing specific lesions, gingivitis correlated with changes in colon and vascular pattern loss, though the association was not statistically significant (*p* = 0.092 and 0.087 respectively). Furthermore, the results of the histological analysis confirmed the association between rectal inflammation and the presence of gingivitis in seven out of eight patients (87.5%).

**Fig. 6:**
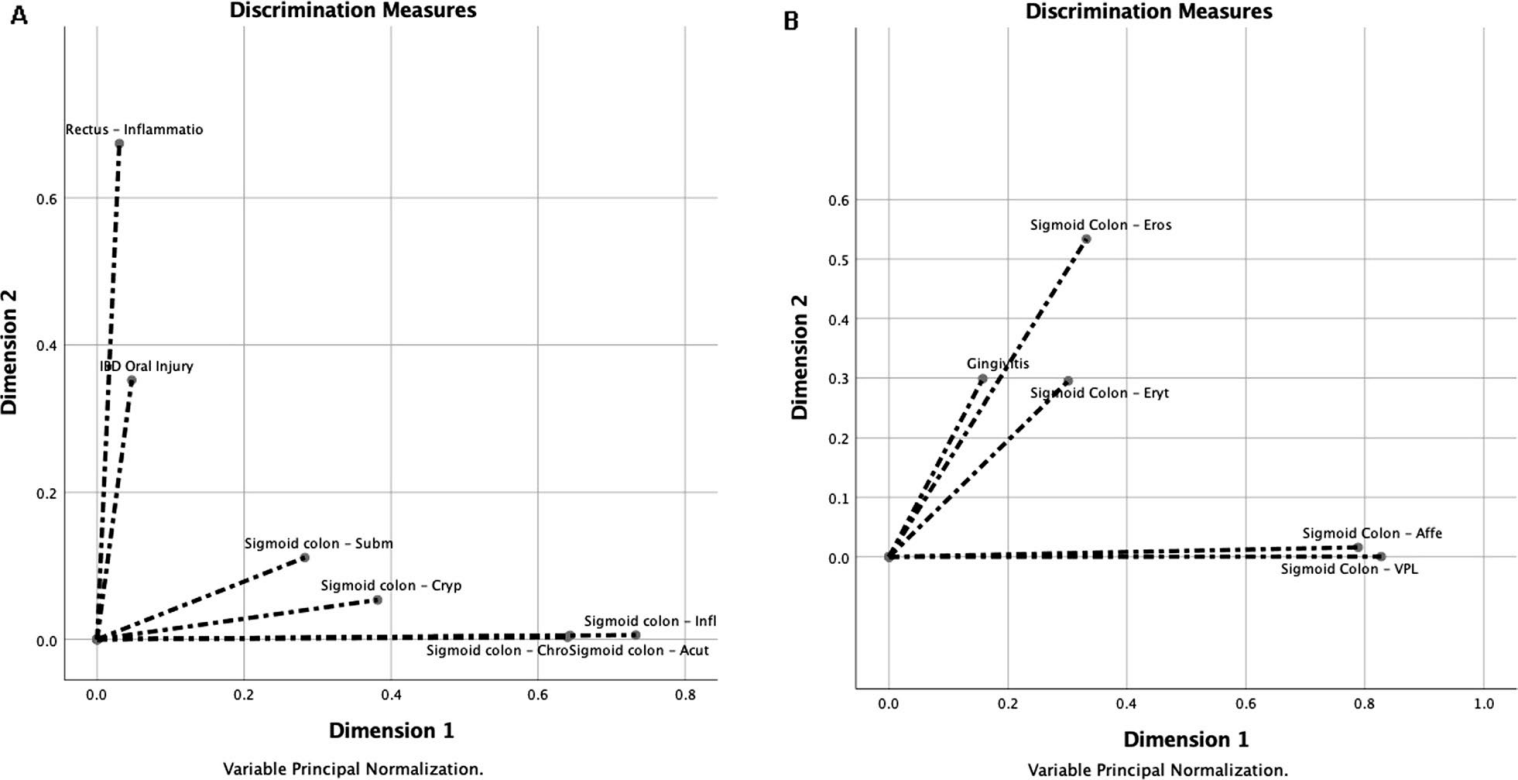
**A** Multiple correspondence discriminant analysis of oral lesions associated with IBD in patients with spondyloarthritis (SpA) and variables obtained through ileocolonoscopy with magnification. The association between the inflammation in the rectum and presence of inflammatory bowel disease–associated oral lesions in patients with SpA. IBD, inflammatory bowel disease; CRYP, cryptitis; SUBM, submucous; CHRO, chronic; ACUT, acute; INFL, inflammation. **B** Multiple correspondence discriminant analysis of oral lesions associated with sigmoid colon in patients with SpA and variables obtained through ileocolonoscopy with magnification. The association between the changes in the colon mucosa and presence of gingivitis in patients with SpA. AFFE, affection; EROS, erosion; ERYT, erythema; VPL, vascular pattern loss

Multiple correspondence discriminant analysis helped show two groups/dimensions, one of which facilitated the observation of the close association between the inflammation of the rectum (CC = 0.674) and presence of lesions in the oral cavity (0.352), with a Cronbach's alpha of 0.578 (Fig. 6A). Likewise, for multiple correspondence discriminant analysis helped also observe a dimension characterized by the presence of gingivitis (CC = 0.300), which was positively associated with the presence of erosions in the sigmoid colon (CC = 0.534) and comparatively less associated with erythematous lesions in the same location (CC = 0.300; Fig. 6B).

Finally, worth mentioning that six out of seven patients, in whom vascular pattern loss was noted in the colon, presented gingivitis in the oral cavity with a significant trend (*p* = 0.092).

## Discussion

It is indisputable that inflammation of the intestine and joints is related to SpA. Thanks to advances in basic research technology and detailed monitoring of well-defined patient cohorts, research in this field is producing data of unprecedented depth. However, the nature of the gut-joint link remains unclear; specifically, the value of intestinal inflammation to predict the development of SpA is still unclear. However, it is a priority to have tools that allow early detection of IBD associated with SpA, impact the quality of life of patients and reduce morbidity and mortality in these patients when both entities are present [23]. Additionally, up to 60.0% of patients with SpA present with subclinical inflammatory bowel involvement, which supports the importance of conducting endoscopic studies and the priority of evaluating associated gastrointestinal symptoms [7, 24, 25].

This supports the importance of evaluating the frequency of gastrointestinal symptoms associated with IBD and early changes in the intestinal and oral mucosa to improve SpA patient referral between oral pathologist, rheumatology and gastroenterology departments.

SpA can manifest itself with extra-articular symptoms in almost all systems and organs in addition to affecting the axial and peripheral skeleton, significantly influencing patients’ quality of life and functional status. The oral cavity is one of the extra-articular sites that could be affected in SpA, especially with gastrointestinal involvement (subclinical spectrum to established IBD) [3].

Men were predominant in the present cohort (56.0%), with a mean age of 42 years. Most patients had AS (82.0%), and 41.0% were active employees, despite this form of SpA is mostly associated with the limitation of patients’ physical activity until rendering them physically disable [26]. In 2012, Casals-Sánchez et al. analyzed a valid sociodemographic data of 1,168 patients with SpA and found AS to be the predominant form. In that cohort, 52.8% of patients had an active working life [27].

As expected, 100.0% patients reported at least one musculoskeletal symptom with the most common being inflammatory low back pain, followed by enthesitis and arthritis. Similarly, regarding the degree of active rheumatological disease, a significant percentage of moderate to high rates were observed in the patients (BASDAI ≥ 4: 52.0%; ASDAS-CRP ≥ 2.1: 74.3%; and ASDAS-ESR ≥ 2.1: 94.0%).

Gastrointestinal manifestations in patients with SpA were relevant in the present study as 69.0% patients presented some gastrointestinal sign or symptom compared with 7.7% healthy controls. In the study conducted in Colombia in 2017 with 102 SpA patients, Romero et al. recorded various gastrointestinal symptoms: abdominal distention (54.9%), abdominal pain (54.9%), diarrhea (34.3%), blood in stool (14.7%), mucus in stool (20.6%), unexplained weight loss (28.4%), and multiple food intolerance (33.4%) [28]. Their findings are very similar to those found in the present Colombian cohort, with most frequent gastrointestinal clinical manifestations associated with IBD being abdominal pain (61.0%), abdominal distention (59.0%), fatigue (59.0%), food intolerance (56.0%), and diarrhea (43.2%).

Furthermore, in the present study, oral lesions were observed in 63.0% patients. In 1991, previous work by Plauth et al. reported 228 oral lesions in 60.0% of 79 patients diagnosed with CD. The lesions included mucosal edema (16.3%), aphthous ulcers and polyps (11.8%) and oral erythema (5.2%) [29]. In 1996, Lisciandrano et al. reported the presence of oral lesions in 32.0% patients with IBD compared with the controls. The predominant lesions were angular cheilitis in patients with CD and UC (7.8% and 5.0% respectively), lichen planus (6.5% and 5.8%, respectively), aphthous ulcers (5.2% and 5.8%, respectively), and candidiasis (5.2% and 0.8%, respectively) [30].

Other studies, such as that by Katz et al. in 2003, explored oral signs, symptoms and overall disease activity in patients with IBD, UC and CD respectively: oral ulceration (25.0% in vs 44.0%), white and hairy tongue (40.0% vs 44.0%), geographic tongue (0.0% vs 15.0%), and fissured tongue (0.0% vs 6.0%) [31]. In 2012, Elahi et al. obtained significant results in 34.0% of 50 patients with UC who presented some oral signs or symptom such as oral ulceration (20%), tongue coating (14%), geographic tongue (2%), fissured tongue (2%), dry mouth (30%) and halitosis (34%) [32]. Furthermore, in 2013, Lankarani et al. reported the presence of aphthous stomatitis in up to 10.0% patients with UC and up to 30.0% patients with CD [33]. On the other hand, in 2016, Tan et al. reported that ulceration or aphthous stomatitis is one most common oral lesion associated with UC (as observed in 30.0% patients). In terms of gingivitis and periodontitis, it occurs in 20.0% patients with UC [34, 35]. Recently, Ribaldone et al. (2020) various reported that oral lesions are frequently observed in patients with IBD, with a prevalence rate of 5.0–50.0% [36].

In the present study, oral lesions were noted in 63.0% patients, similar to Plauth et al. who reported 60.0% [29]. This percentage is practically twice as much as those reported by Lisciandrano et al. and Elahi et al. (32.0% and 34.0%, respectively) [30, 32]. To our knowledge, we have not found previous reports of oral manifestations associated with IBD in patients with SpA who do not have a concomitant diagnosis of IBD. However, it is interesting to contrast with the oral findings in IBD given the strong relationship between the two entities.

In the present study, the IBD-associated lesions detected were gingivitis (55.0%, which is statistically significant; therefore, its evaluation is crucial), followed by aphthous stomatitis (3.8%), angular cheilitis (2.6%), and perioral erythema with desquamation (1.3%). Gingivitis is defined as acute inflammation of the gums with vascular changes of vascular dilation and increased blood circulation, product of bacterial or systemic factors in gingival tissues [37].

Gingivitis was similarly reported by Jurge et al. in 64.0% patients, whereas Plauth et al. and Tan et al. in < 20.0% [29, 34, 38]. On the basis of the International Workshop for the Classification of Periodontal Diseases and Conditions of 2017, gingivitis affecting the patients in the present study can be classified within the conditions and periodontal diseases that affect the periodontal support tissues due to systemic diseases that are not gingivitis induced by biofilm or dental plaque [39]. These are considered granulomatous inflammatory conditions or conditions that can generate orofacial granulomatosis such as CD, which is directly related to our patients [40]. The high general disease burden might affect oral health impact profile, making an increased attention of these patients in dental care, especially considering psychological aspects, necessary [41].

It is also important to consider the effect of treatment on oral lesions involves different medications such as: 5 amino salicylic acid derivatives, corticosteroids, immunomodulators, calcineurin inhibitors, biological therapy and antibiotics; the selection of treatment will depend on the site where the disease is affecting, and its course or behavior [42–44]. Oral complications associated with the use of these treatments were excluded since are not used in this type of disease. However, since 60% of patients were treated with biological therapy, the oral cavity may act as a bacterial reservoir leading to unwanted local or systemic complications. Bacterial residues can promote local inflammation on the gingival tissue [45]. Of the patients treated with biological therapy, 54.2% presented a diagnosis of gingivitis. Which has been described in other pathologies such as rheumatoid arthritis [46].

Compared with the literature, aphthous stomatitis/oral ulceration was noted less frequently in the present study; other studies have reported percentages between 5.2% and 87.0% [29–36, 38]. However, Sanz Sanz et al. only included aphthous stomatitis as an oral sign in the screening criteria for the referral of patients to the gastroenterology department from the rheumatology department. Thus, this finding on oral clinical evaluation is crucial because the patient can be referred to the gastroenterology department and IBD can be diagnosed early [47].

Angular cheilitis was reported in 7.8–67.0% patients with IBD in other studies [30, 38]; however, only 2.6% patients in the present study presented angular cheilitis. On the other hand, perioral erythema was reported by Plauth et al. in only 5.2% patients, and its occurrence was very low in the present cohort [29], which may reflect characteristics of our miscegenation and environment.

Other lesions frequently found among patients with UC and CD, such as lichen planus, candidiasis, white and hairy tongue, geographic tongue, and fissured tongue have been reported by other studies; however, these were not detected in the present cohort [32, 48]. In 2016, Vasovic et al. reported that the oral symptoms of CD precede intestinal manifestations in 60.0% patients and, in many cases, aid in the diagnosis of the disease [49]. In addition, active CD is associated with more oral signs in 70.6% patients compared with only 52.1% of patients in remission [31], with aphthous ulcers being the most common type of mucosal lesion [50].

After evaluating the findings of the colonoscopic analysis with digital chromoendoscopy and magnification of the sigmoid colon mucosa, 100% patients who presented changes in the colonic mucosa were found to have some oral lesion significantly associated with IBD, predominantly aphthous gingivitis/stomatitis. Of note, the results of the histological analysis supported the association between the inflammation of the rectum and presence of gingivitis.

Although the manifestations of CD in the oral cavity may precede intestinal manifestations or may occur at the same time or after intestinal manifestations, oral lesions can be important in the early diagnosis of IBD in patients with SpA. This condition can be easily confirmed by a histopathological analysis of the lesions in the oral cavity [51, 52].

The major limitation of this study was that the participants were included at a time that did not allow for follow-up and evaluation of their lesions; however, they were referred to the gastroenterology department in a timely manner.

## Conclusions

The oral lesions were associated with symptoms of IBD in patients with SpA without a confirmed diagnosis of IBD. Clinical oral signs and symptoms were general gingivitis, aphthous stomatitis, angular cheilitis, and perioral erythema with scaling, being the first two important due to their identified association with IBD and included in the screening criteria for early symptoms of IBD. Patients with SpA and gastrointestinal symptoms without a diagnosis of IBD have more oral signs and symptoms than healthy controls. Hence, clinical evaluation by oral pathologists/oral physicians is essential in the early diagnosis of this disease. Comprehensive and multidisciplinary management paired with the gastroenterological and rheumatological evaluations is of vital importance to improve the quality of life of patients with SpA in early stages.

## Supplementary Information

**Additional file 1.** Sociodemographic Characteristics of Patients and Healthy Controls.

## Data Availability

The datasets generated and/or analyzed during the current study are not publicly available due to the privacy of the individuals who participated in the study, but are available from the corresponding author on reasonable request.
