# Supplementary material for: Oral manifestations associated with inflammatory bowel disease and early endoscopic findings in patients with spondyloarthritis
Source: BMC Oral Health. 2022 Nov 9;22:477. doi: 10.1186/s12903-022-02497-4 (PMC9644594; doi:10.1186/s12903-022-02497-4)
Supplement: Supplementary file 1 — Additional file 1. Sociodemographic Characteristics of Patients and Healthy Controls. [file 12903_2022_2497_MOESM1_ESM.docx]

**SUPPLEMENTARY TABLE 1. SOCIODEMOGRAPHIC CHARACTERISTICS OF PATIENTS AND HEALTHY CONTROLS.**

| **CHARACTERISTIC** | **PATIENTS**  **(N=80)** | | **HEALTHY CONTROLS**  **(N=52)** | |
| --- | --- | --- | --- | --- |
|  | **n** | **%** | **n** | **%** |
| **Age*** | 42,8 ± 10,4 | | 41,0 ± 13,6 | |
| **Sex** |  | |  | |
| Female | 35 | 44,0 | 28 | 54,0 |
| Male | 45 | 56,0 | 24 | 46,0 |
| **Active smoker***** | 5 | 6,2 | 8 | 15,0 |
| **Smoking history***** | 25 | 31,0 | 16 | 31,0 |
| **Passive smoker***** | 12 | 15,0 | 11 | 21,0 |
| **Occupational status** |  | |  | |
| Homemaker | 12 | 15,0 | 2 | 3,8 |
| Independent | 20 | 25,0 | 3 | 5,7 |
| Employee | 33 | 41,0 | 40 | 77,0 |
| Pensioner | 11 | 14,0 | 4 | 7,7 |
| Student | 4 | 5,0 | 3 | 5,8 |
| **Living place** |  | | | |
| Own | 41 | 51,0 | 35 | 6,7 |
| Leased | 27 | 34,0 | 17 | 33,0 |
| Common | 9 | 11,0 | 0 | 0,0 |
| Accommodation | 3 | 4,0 | 0 | 0,0 |
| **Marital status** |  | |  | |
| Married | 45 | 56,0 | 19 | 37,0 |
| Single | 17 | 21,0 | 21 | 40,0 |
| Widowed | 16 | 20,0 | 11 | 21,0 |
| Common law marriage | 2 | 3,0 | 1 | 2,0 |
| **Education level** |  | |  | |
| Primary | 1 | 1,0 | 2 | 4,0 |
| High school | 28 | 35,0 | 18 | 34,0 |
| Technical school | 12 | 15,0 | 4 | 8,0 |
| University | 39 | 49,0 | 28 | 54,0 |
| **BMI**** | 25,6±3,6 | | 25,1±3,6 | |
| **BMI >25** | 48 | 52,0 | 25 | 49,0 |
| *Data expressed as mean ± SD  **Data expressed as median (interquartile range)  ***Affirmative at asking | | | | |
